# Estimation of Tri-Axial Walking Ground Reaction Forces of Left and Right Foot from Total Forces in Real-Life Environments

**DOI:** 10.3390/s18061966

**Published:** 2018-06-19

**Authors:** Erfan Shahabpoor, Aleksandar Pavic

**Affiliations:** 1 Department of Architecture and Civil Engineering, University of Bath, Claverton Down, Bath BA2 7AY, UK; 2 Department of Civil & Structural Engineering, INSIGNEO Institute for In-Silico Medicine, University of Sheffield, Sheffield S1 3JD, UK; 3 College of Engineering, Mathematics and Physical Sciences, University of Exeter, Exeter EX4 4QF, UK; a.pavic@exeter.ac.uk

**Keywords:** *GRF*, polynomial, curve fitting, double support, closed kinematic chain, indeterminacy problem

## Abstract

Continuous monitoring of natural human gait in real-life environments is essential in many applications including disease monitoring, rehabilitation, and professional sports. Wearable inertial measurement units are successfully used to measure body kinematics in real-life environments and to estimate total walking ground reaction forces GRF(t) using equations of motion. However, for inverse dynamics and clinical gait analysis, the GRF(t) of each foot is required separately. Using an experimental dataset of 1243 tri-axial separate-foot GRF(t) time histories measured by the authors across eight years, this study proposes the ‘Twin Polynomial Method’ (TPM) to estimate the tri-axial left and right foot GRF(t) signals from the total GRF(t) signals. For each gait cycle, TPM fits polynomials of degree five, eight, and nine to the known single-support part of the left and right foot vertical, anterior-posterior, and medial-lateral GRF(t) signals, respectively, to extrapolate the unknown double-support parts of the corresponding GRF(t) signals. Validation of the proposed method both with force plate measurements (gold standard) in the laboratory, and in real-life environment showed a peak-to-peak normalized root mean square error of less than 2.5%, 6.5% and 7.5% for the estimated GRF(t) signals in the vertical, anterior-posterior and medial-lateral directions, respectively. These values show considerable improvement compared with the currently available GRF(t) decomposition methods in the literature.

## 1. Introduction

The tri-axial walking ground reaction forces, moments and the trajectory of center of plantar pressure CoP(t) under each foot are critical inputs for gait analysis [1]. Regardless of their importance, developing practical technologies for long-term measurement of these parameters in real-life environment are still challenging.

Estimation of the total (superimposed left and right foot) walking $GRF_{i}\left(t\right)$, i∈{v: vertical,ap:anterior−posterior,ml:medial−lateral} using a limited number of inertial measurement units (IMUs) is a practical option to monitor the total $GRF_{i}\left(t\right)$ in real-life environments [2,3] due to the high durability, low power demand, low cost, and small size of the IMUs. In this method, the total $GRF_{i}\left(t\right)$ signals are often estimated based on the Second Newton law using the measured acceleration of the body segments. During the single-support (SS) phase of the gait, $GRF_{i}\left(t\right)$ of the stance foot is equal to the estimated total $GRF_{i}\left(t\right)$ signals. However, during the double-support (DS) phase, both feet are in contact with the ground, which creates an indeterminate closed kinematic chain [4]. As a result, the left and right foot $GRF_{i}\left(t\right)$ signals cannot be determined from the force equilibrium conditions alone, and an additional mathematical or statistical method is required to solve the indeterminacy problem.

Several methods have been proposed to estimate the left and right foot $GRF_{v}\left(t\right)$ signals from the total $GRF_{v}\left(t\right)$ during the DS phase, based on the weight shift between legs [5], optimization approaches [6], or the plantar center of pressure [7]. Quanbury and Winter [8], and Robertson and Winter [9] suggested extrapolating the total $GRF_{v}\left(t\right)$ during the DS phase by fitting cubic polynomials to the known force values at the beginning and end of the SS phase for each foot. They reported normalized root mean square error of 18% against force plate measurement for the estimated left and right foot $GRF_{v}\left(t\right)$ signals. McGhee et al. [10], McGhee [11], Hardt and Mann [12], and Morecki et al. [13] suggested a linear function of the DS phase time to approximate the force and moment transfer between the stance and swing foot. Their proposed method estimated the left and right foot $GRF_{v}\left(t\right)$ signals with maximum normalized error of 8%.

In 2005, Ren et al. [14] initially proposed a linear transfer relationship based on empirical data. They assumed that the ratio of the $GRF_{v}\left(t\right)$ of the heel-strike foot to the total $GRF_{v}\left(t\right)$, and the ratio of the $GRF_{ap}\left(t\right)$ to the $GRF_{v}\left(t\right)$ on the toe-off foot vary linearly during the DS phase. It was also assumed that the right and left foot move symmetrically. Later in 2008, Ren et al. [15] proposed the “smooth transition assumption” (STA), based on force plate data for each foot:

1. The ground reaction forces and moments of the trailing foot reduce smoothly to zero during the DS phase.
2. The ratios of the ground reactions to their values at contralateral heel strike (i.e., the non-dimensional ground reactions) can be expressed as functions of DS phase duration (termed transition functions).

The model proposed by Ren et al. [15] showed good results in the sagittal plane, but results were less promising in the frontal and transverse planes. They reported 5.6%, 10.9% and 20% relative root mean square error for estimation of separate feet $GRF_{v}\left(t\right)$, $GRF_{ap}\left(t\right)$, and $GRF_{ml}\left(t\right)$, respectively. Subsequently, several studies were carried out to increase the accuracy of the estimated left and right foot $GRF_{v}\left(t\right)$ [16,17,18,19]. However, the proposed methods in these studies require the trajectory of CoP(t) as input and, therefore, are mostly suitable for force plate application.

Recently, Karatsidis et al. [20] proposed a comprehensive methodology to estimate tri-axial $GRF_{i}\left(t\right)$ and moment signals for each foot using inertial motion capture data. The total external forces and moments were calculated directly from equation of motion. A distribution algorithm based on a smooth transition assumption was then proposed to solve the indeterminacy problem during the DS phase. They reported relative root mean square error of 5.3%, 9.4% and 13.1% for the estimated $GRF_{v}\left(t\right)$,$\;GRF_{ap}\left(t\right)$ and $GRF_{ml}\left(t\right)$, respectively, compared with the force plate measurements. An extensive review of the literature on estimation of walking ground reactions in real-life environments is presented in [21].

This study is aimed at developing a methodology to estimate the tri-axial left and right foot $GRF_{i}\left(t\right)$ from the corresponding total $GRF_{i}\left(t\right)$ signal with: (1) enhanced accuracy; (2) enhanced versatility (applicable to different walking speeds and step lengths); (3) extensively validated and (4) suitable for IMU application i.e., only total $GRF_{i}\left(t\right)$ signals are available as input. To achieve these objectives, a uniquely extensive experimental dataset of 1243 tri-axial left and right foot $GRF_{i}\left(t\right)$ time histories with different walking speeds were used to develop the ‘Twin Polynomial Method’ (TPM) to estimate the tri-axial left and right foot $GRF_{i}\left(t\right)$ from the corresponding total $GRF_{i}\left(t\right)$ signal. Section 2 of this paper explains the TPM methodology and specifications. TPM procedure is presented in Section 3.1 and is validated in both laboratory and outdoor environment in Section 3.2. Section 4 discusses the results and compares the performance of TPM with other methods in the literature. Finally, the conclusions are highlighted in Section 5.

## 2. TPM Methodology

The Twin Polynomial Method proposed in this study assumes that the total $GRF_{i}\left(t\right)$ signals (estimated using a wearable IMU system [21]) and the weight of the subject are the only known inputs to the model and the left and right foot $GRF_{i}\left(t\right)$ signals are the desired outputs. 

**Figure 1:**
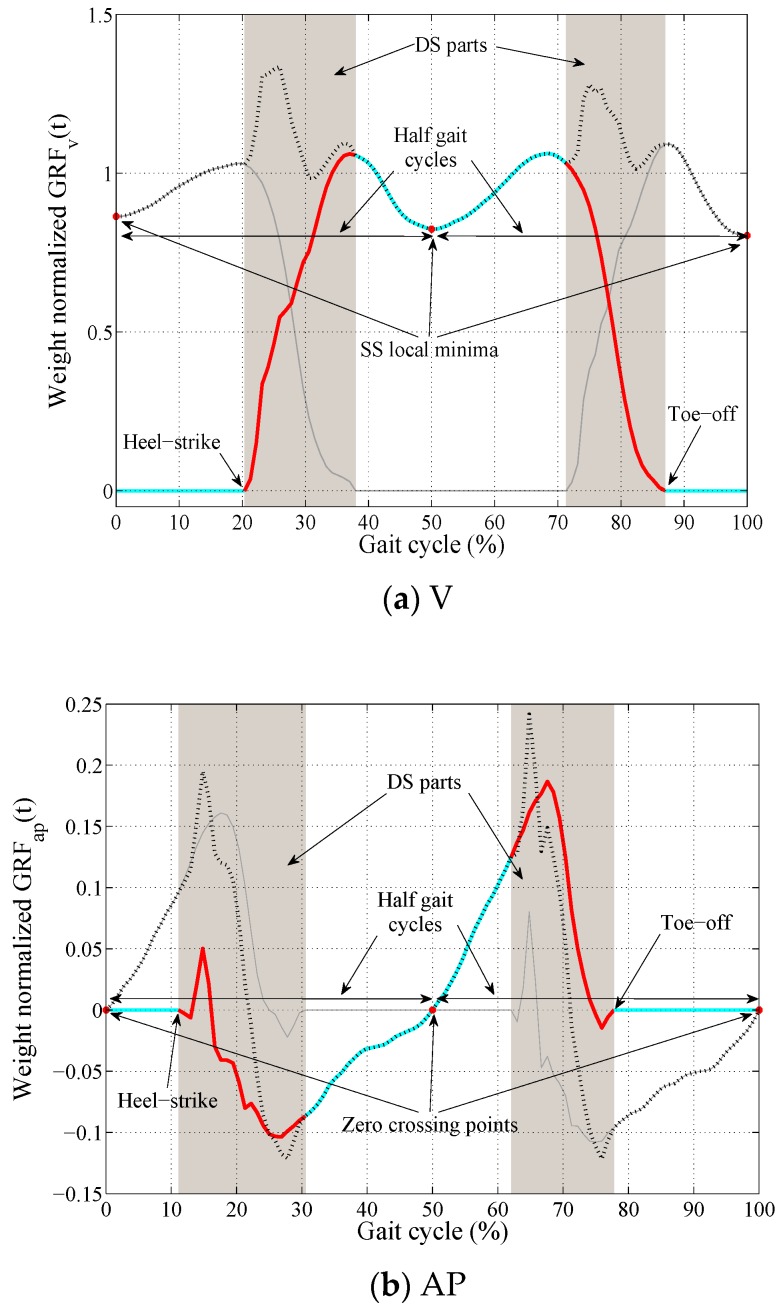

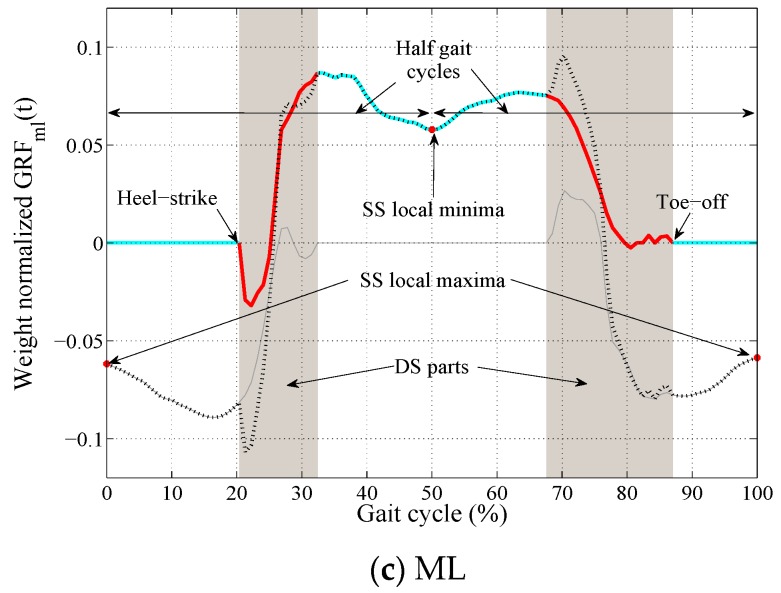
A typical illustration of total (dashed black) and a single foot (Red and blue) $GRF_{v}\left(t\right)$ (**a**), $GRF_{ap}\left(t\right)$ (**b**) and $GRF_{ml}\left(t\right)$, (**c**) during a complete gait cycle.

As mentioned in Section 1, during the SS phase of the gait, only the stance foot is in contact with the ground. Therefore, the magnitude of the stance foot walking force (blue curves in Figure 1) is equal to the total $GRF_{i}\left(t\right)$ (dashed black curves in Figure 1) and the amplitude of the walking force of the swing foot is equal to zero. The challenge is, however, to estimate the left and right foot $GRF_{i}\left(t\right)$ during the DS phase (red curves in Figure 1). The core idea of TPM is to fit a polynomial curve of degree n to the known SS part of each of the left and right foot $GRF_{i}\left(t\right)$ signals (blue curve in Figure 1) to extrapolate the unknown DS part of the corresponding signals (red curves in Figure 1).

To simplify the estimation process, TPM divides each complete gait cycles into two halves and estimates the unknown DS parts of the left and right foot $GRF_{i}\left(t\right)$ signals for each half gait cycle, separately (Figure 1). In the vertical direction, the window of the total $GRF_{v}\left(t\right)$ signal between the two consecutive SS local minima is taken equal to half of a complete gait cycle (Figure 1a). Similarly, in the AP direction, the window of the total $GRF_{ap}\left(t\right)$ signal between two consecutive zero crossing points (Figure 1b), and in ML direction the window of the total $GRF_{ml}\left(t\right)$ signal between the two consecutive SS local minima and maxima points (Figure 1c) are taken equal to half of a complete gait cycle. The left and right foot $GRF_{i}\left(t\right)$ signals corresponding to half of a gait cycle are referred to as $hGRF_{i,j}\left(t\right)$, where i∈{v,ap,ml} indicates the axis and j∈{l,r} represents the left and right part of the half gait force signal, respectively.

### 2.1. Experimental Data

As the walking force is characterized with high inter- and intra-subject variability, a large dataset of measured walking force was required for the analysis to represent these variations statistically. A set of 1243 measured right and left foot walking $GRF_{i}\left(t\right)$ time histories, each lasting between 60 and 240 s and measured from over 200 different test subjects (age: 26 ± 8 years, weight: 77 ± 26 kg, height: 1.74 ± 0.21 m, and walking speed: 1.22 ± 0.44 m/s) with healthy gait were used in the analysis. Measurements were carried out using a bespoke separate-belt instrumented treadmill at 1 kHz between 2008 and 2016 in the biomechanics laboratory at the University of Sheffield. The investigations were carried out following the rules of the Declaration of Helsinki of 1975 (https://www.wma.net/what-we-do/medical-ethics/declaration-of-helsinki/), revised in 2008. Ethics approval for the walking tests was issued by the ethics committee of the Faculty of Engineering, University of Sheffield, and all subjects provided an informed consent in accordance with the University of Sheffield ethical guidelines for research involving human participants.

**Figure 2:**
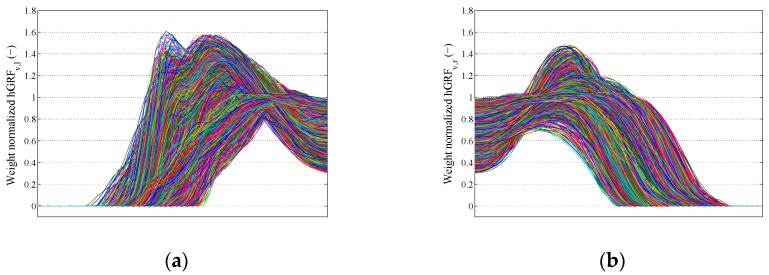
Overlaid plot of $hGRF_{v,l}\left(t\right)$ (**a**) and $hGRF_{v,r}\left(t\right)$ (**b**) signals.

For each of the measured left and right foot $GRF_{i}\left(t\right)$ signals, the heel-strike and toe-off points corresponding to each gait cycle were identified (Figure 1), and the signal corresponding to each half gait cycle was extracted. To make cross-comparison and statistical analysis possible, each $hGRF_{i,j}\left(t\right)$ signal was then resampled to 100 points and normalized by the body weight of the test subject using MATLAB software [22]. This resulted in a dataset of over 100,000 of $hGRF_{i,j}\left(t\right)$ signals. The overlaid plot of one foot $hGRF_{v,l}\left(t\right)$ and $hGRF_{v,r}\left(t\right)$ signals are presented in Figure 2a,b, respectively. As signals are resampled, the variable “*t*” in $hGRF_{i,j}\left(t\right)$ signals does not represent the actual timing of the measured $hGRF_{i,j}\left(t\right)$ signals any more. However, the instances of the resampled $hGRF_{i,j}\left(t\right)$ signals are still referred to by the variable “*t*” in this study, indicating their nature.

### 2.2. TPM Specifications

**Figure 3:**
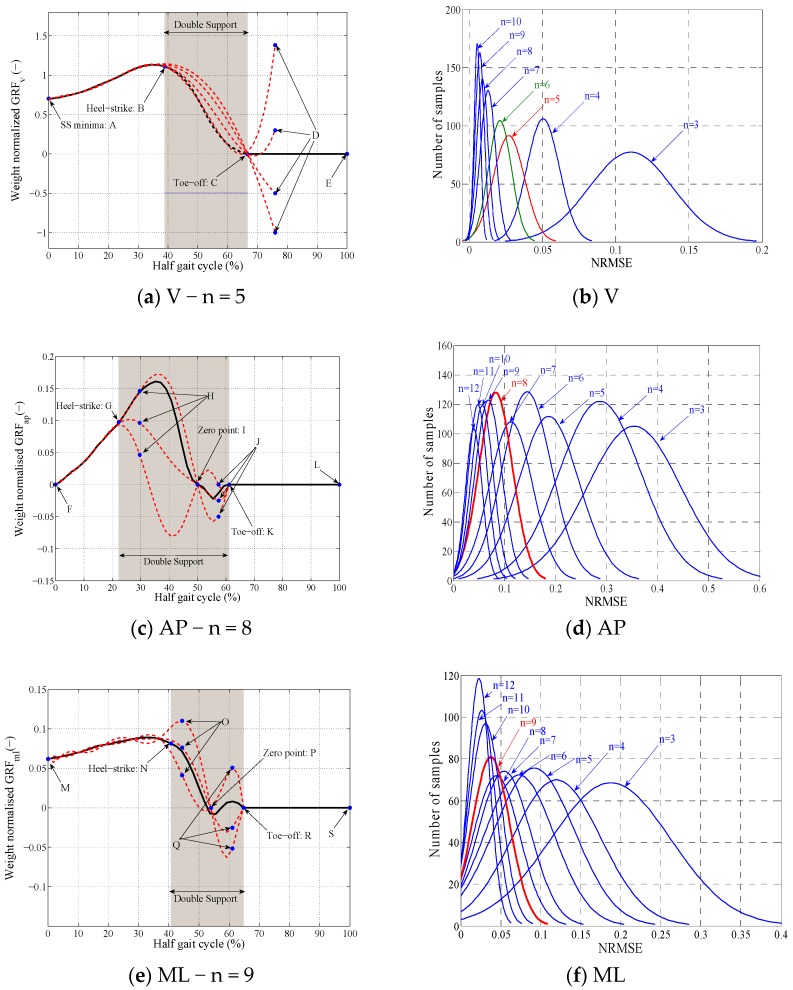
Using polynomials of degree *n* to fit $hGRF_{v}\left(t\right)$ signals.

In order to estimate the unknown DS part of the $hGRF_{i,j}\left(t\right)$ signals, TPM proposes to fit a polynomial curve of degree n to the known SS part of the $hGRF_{i,j}\left(t\right)$ signals (e.g., A–B in Figure 3a) and their corresponding zero point (e.g., point C in Figure 3a). In this study, the MATLAB [22] function ‘polyfit’ was used for curve fitting which uses Vandermonde matrix to estimate the coefficients of the best polynomial fit in a least-square sense.

#### 2.2.1. Identification and Validation Experimental Datasets

The measured $hGRF_{i,j}\left(t\right)$ signals corresponding to 20 randomly-selected subjects were initially extracted from the experimental dataset to be used only for model validation. The remainder of the dataset was used for model identification. As the $hGRF_{i,j}\left(t\right)$ dataset was very large, for practicality, it was necessary to use a smaller sub-set of it for model identification which statistically represents the main dataset. To find the size of this subset, a Monte Carlo analysis was carried out with increasing number of measured $hGRF_{i,j}\left(t\right)$ signals fitted with polynomials of degree three (cubic) to 12. Each randomly selected $hGRF_{i,j}\left(t\right)$ signal from the experimental dataset, was fitted with a polynomial $P_{n}\left(t\right)$ of degree *n*, and the corresponding peak-to-peak normalized root mean square error (NRMSE), defined by Equation (1), was calculated:

(1) $$\;\mathrm{NRMSE}=\frac{\sqrt{\left(\sum_{t=1}^{100}{\left(hGRF_{i,j}\left(t\right)-P_{n}\left(t\right)\right)}^{2}\right)/100}}{\left|\max\left(hGRF_{i,j}\left(t\right)\right)-\min\left(hGRF_{i,j}\left(t\right)\right)\right|}$$

In Equation (1), the values of $P_{n}\left(t\right)$ when the foot is in swing is set to zero. 

It was found that a subset of minimum 940 $hGRF_{i,j}\left(t\right)$ randomly-selected signals can represent statistically the whole dataset in all three axes with less than 1% NRMSE standard deviation $\;\sigma_{NRMSE}$. Therefore, a set of 1000 $hGRF_{i,j}\left(t\right)$ randomly-selected signals was used in Section 2.2.2 to Section 2.2.4 to develop TPM.

#### 2.2.2. Polynomial Degree Selection

To find the optimal polynomial order that can fit accurately the $hGRF_{i,j}\left(t\right)$ signals, in each direction, each of the N = 1000 $hGRF_{i,j}\left(t\right)$ randomly-selected measured signals was fitted with polynomials of degree three (cubic) to 12. Figure 3 compares the probability distribution function (pdf) of the NRMSE errors corresponding to each polynomial degree. There is a tradeoff between the fit accuracy and the computational efficiency of the fitting process: the higher the polynomial order of the fitting curve is, the more computationally demanding and challenging would be to find the best fit, but a more accurate fit can potentially be achieved. 

Based on Figure 3, polynomials of degree five, eight, and nine were selected empirically to fit the $hGRF_{i,j}\left(t\right)$ signals in the vertical, anterior-posterior, and medial-lateral directions, respectively. The choice was made to maximize the accuracy of the fit while avoiding unnecessary computation. As it can be seen in Figure 3, in each direction, the reduction in NRMSE of polynomials with lower orders than V:5, AP:8 and ML:9 is significant and for the higher orders is subtle.

#### 2.2.3. Added Constraints

As it can be seen in Figure 3a,c,e, when only the SS part of the $hGRF_{i,l}\left(t\right)$ and $hGRF_{i,r}\left(t\right)$ signals and their zero point are known, several polynomial fits of degree five (Figure 3a), eight (Figure 3c), and nine (Figure 3e) exist. In another word, the SS part and zero point are not enough constraints to produce a unique polynomial fit of degree five, eight, and nine to $hGRF_{v,j}\left(t\right)$, $hGRF_{ap,j}\left(t\right)$, and $hGRF_{ml,j}\left(t\right)$ signals, respectively.

Using Monte Carlo analysis, it was found that one extra (guiding) point in the vertical direction (point D in Figure 3a), three points in the AP direction (points H, I and J in Figure 3c), and three points in the ML direction (points O, P and Q in Figure 3e) are enough to achieve polynomial fits with maximum 1% difference in $\mu_{NRMSE}$ and $\sigma_{NRMSE}$ compared with the polynomials in Figure 3b,d,f. Polynomials of higher order failed to achieve comparable $\mu_{NRMSE}$ and $\sigma_{NRMSE}$ values with the same number of guiding points (2–5% higher error).

**Figure 4:**
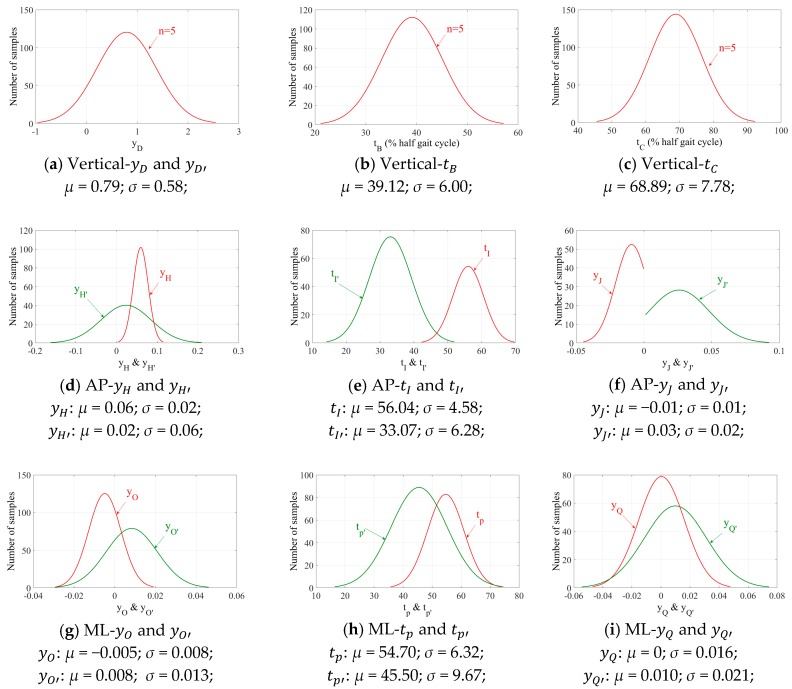
The normal distribution fit representing polynomials’ parameters.

The magnitude (y) and timing (t) of the guiding points of the best polynomial fits, were statically analyzed (Figure 4) for the random sample of N = 1000 $hGRF_{i,j}\left(t\right)$ signals to find out the statistical distribution of $t_{B}$, $t_{C}$, $y_{D}$, $y_{H}$, $t_{I}$, $y_{J}$, $y_{O}$, $t_{P}$, and $y_{Q}$ parameters (and $y_{D^{\prime }}$, $y_{H^{\prime }}$, $t_{I^{\prime }}$, $y_{J^{\prime }}$, $y_{O^{\prime }}$, $t_{P^{\prime }}$ and $y_{Q^{\prime }}$ for the counterpart $hGRF_{i,j}\left(t\right)$ signals) suitable for curve-fitting analysis (Table 1). It was empirically assumed that $t_{D}=t_{C}+0.1T$, $t_{H}=t_{G}+0.05T$, $t_{J}=\left(t_{I}+t_{k}\right)/2$, $t_{O}=t_{N}+0.05T$ and $t_{Q}=\left(t_{P}+t_{R}\right)/2$. The range of μ±2σ (representing 95% of the $hGRF_{i,j}\left(t\right)$ signals) corresponding to each of these parameters (Figure 4) are used in TPM curve-fitting procedure as explained in Section 3 (Table 1).

#### 2.2.4. Optimization Strategy

As it can be seen in Figure 3a,c,e, a family of polynomial fits can be generated using different guiding points that match the known SS part of the $hGRF_{i,j}\left(t\right)$ signal while they do not necessarily match the DS part of the $hGRF_{i,j}\left(t\right)$ signal. The strategy proposed by TPM to circumvent this problem is to use the fitted polynomials on both the $hGRF_{i,l}\left(t\right)$ (Figure 3a,c,e) and $hGRF_{i,r}\left(t\right)$ signals and compare their total curve with the known total $hGRF_{i}\left(t\right)$ for that half gait cycle. This ensures that the information available in the total $hGRF_{i}\left(t\right)$ signal during the DS phase is optimally used to find the pair of polynomial curves that best fit the $hGRF_{i,l}\left(t\right)$ and $hGRF_{i,r}\left(t\right)\;$ signals. Section 3 describes the TPM procedure in details.

## 3. Results

### 3.1. TPM Procedure

The step-by-step procedure proposed by TPM to estimate $hGRF_{i,l}\left(t\right)$ and $hGRF_{i,r}\left(t\right)$ from the known total $hGRF_{i}\left(t\right)$ is:

*A.*  Vertical direction

**Figure 5:**
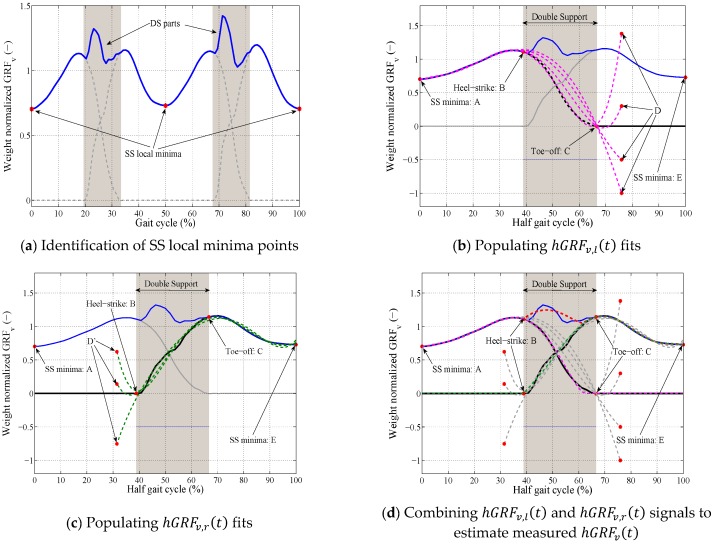

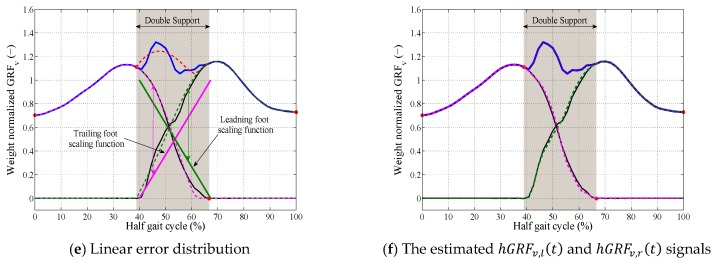
TPM procedure for estimation of $hGRF_{v}\left(t\right)$.

- In the first step, the SS local minima points are identified from the total $GRF_{v}\left(t\right)$ (Figure 5a).
- For each segment of the total $GRF_{v}\left(t\right)$ signal between two consecutive local minima (total $hGRF_{v}\left(t\right)$):
  - The total $hGRF_{v}\left(t\right)$ segment is resampled to 100 points (*T* = 100) and is normalized to the weight of the subject (Figure 5b).
  - For each pair of toe-off ($t_{C}$) and heel-strike ($t_{B}$) points selected from their initial ranges (27 < $\;t_{B}<$ 52 and 53 < $\;t_{C}<$ 85):
    - A set of polynomial curves of degree five are generated using the SS part (A-B) and points C and D for the $hGRF_{v,l}\left(t\right)$ (Figure 5b), and E-C, B, and $D^{\prime }$ for the $hGRF_{v,r}\left(t\right)\;$ (Figure 5c) where −1 < $y_{D}\;\mathrm{and}\;y_{D^{\prime }}$ < 3.5, $t_{D}=\;t_{C}+0.1T$ and $t_{D^{\prime }}=\;t_{B}-0.1T$ (Table 1).
    - A pair of left (pink curve in Figure 5d) and right (green curve in Figure 5d) polynomial curves are found to represent this $t_{C}$, $t_{B}$ combination that their total curve (red curve in Figure 5d) estimated the total $hGRF_{v}\left(t\right)$ with minimum NRMSE.
    - The procedure is repeated for all possible combinations of $t_{C}$ and $t_{B}$ selected from their initial ranges (Table 1).
  - The pair of left-right polynomial curves that estimated the total $hGRF_{v}\left(t\right)$ with minimum NRMSE is found. These curves are considered accurate estimates of the $hGRF_{v,l}\left(t\right)$ and $hGRF_{v,r}\left(t\right)$ signals for the current half gait cycle.
  - The difference between the measured (blue curve in Figure 5e) and estimated total $\;hGRF_{v}\left(t\right)$ (dashed red curve in Figure 5e) curves during the DS phase $Err_{v}\left(t\right)$, are distributed between the estimated $hGRF_{v,l}\left(t\right)$ (dashed pink curve in Figure 5e) and $hGRF_{v,r}\left(t\right)$ (dashed green curve in Figure 5e) signals using Equations (3) and (4) when $t_{B}<t<t_{C}$ and i=v:
    (2) $$\;Err_{i}\left(t\right)=\;measured\;total\;hGRF_{i}\left(t\right)-estimated\;total\;hGRF_{i}\left(t\right)$$
    (3) $$\;hGRF_{i,l}\left(t\right)=hGRF_{i,l}\left(t\right)+S_{l}\left(t\right)\times Err_{i}\left(t\right)$$
    (4) $$\;hGRF_{i,r}\left(t\right)=hGRF_{i,r}\left(t\right)+S_{r}\left(t\right)\times Err_{i}\left(t\right)$$
    In these equations, the linear scaling functions $S_{l}\left(t\right)$ (pink line in Figure 5e) and $S_{r}\left(t\right)$ (green line in Figure 5e) are empirically defined as:
    (5) $$\;S_{l}\left(t\right)=\frac{t-t_{B}}{t_{C}-t_{B}}$$
    (6) $$\;S_{r}\left(t\right)=\frac{t_{B}-t}{t_{C}-t_{B}}+1$$
  - The estimated $hGRF_{v,l}\left(t\right)$ and $hGRF_{v,r}\left(t\right)$ signals (dashed curves in Figure 5f) are multiplied by the weight of the subject and resampled back to the actual length of the measured total $\;hGRF_{v}\left(t\right)$ signal for the current half gait cycle.
- The next half gait cycle is selected and the estimation procedure described in Step A.II is repeated.

*B.*  *Anterior-posterior direction*

**Figure 6:**
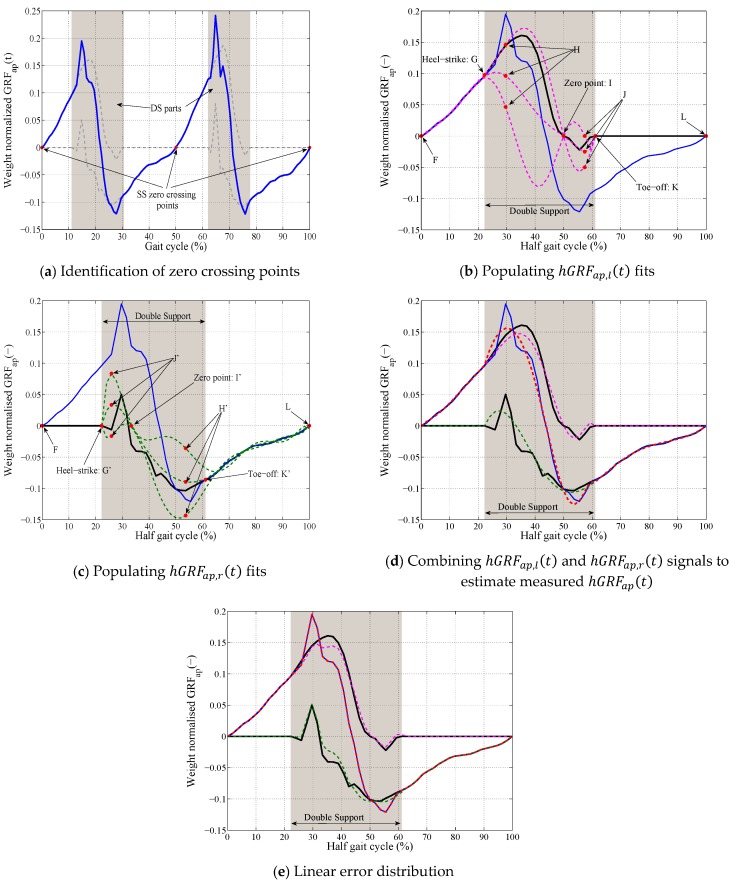
TPM procedure for estimation of $hGRF_{ap}\left(t\right)$.

- In the first step, the SS phase zero crossing points are identified from the total $GRF_{ap}\left(t\right)$ (Figure 6a).
- For each segment of the total $GRF_{ap}\left(t\right)$ signal between two consecutive zero crossing points (total $hGRF_{ap}\left(t\right))$:
  - The total $hGRF_{ap}\left(t\right)$ segment is resampled to 100 points (*T* = 100) and normalized to the weight of the subject (Figure 6b).
  - The timing of the heel-strike ($t_{G}$) and toe-off ($t_{K}$) points of this half gait cycle are identified from the previously-estimated $hGRF_{v,r}\left(t\right)$ and $hGRF_{v,l}\left(t\right)$ signals corresponding to this half gait cycle.
  - A set of polynomial curves of degree eight are generated using the SS part (F-G) and points H, I, J and K for the $hGRF_{ap,l}\left(t\right)$ (Figure 6b) where $t_{H}=t_{G}+0.05T$, $t_{J}=\left(t_{I}+t_{k}\right)/2$, $0.02<y_{H}<\;0.10$, $-0.02<y_{J}<0$ and $0.46T<t_{I}<0.65T$ (Table 1).
  - A set of polynomial curves of degree eight are generated using the SS part L-$K^{\prime }$, $H^{\prime }$, $I^{\prime }$, $J^{\prime }$ and $G^{\prime }$ for the $hGRF_{ap,r}\left(t\right)\;$ (Figure 6c) $t_{H^{\prime }}=t_{K^{\prime }}-0.05T$, $t_{J^{\prime }}=\left(t_{I^{\prime }}+t_{G^{\prime }}\right)/2$, $-0.10<y_{H^{\prime }}<\;0.14$, $-0.01<y_{J^{\prime }}<0.07$ and $0.20T<t_{I^{\prime }}<0.46T$ (Table 1).
  - A pair of left (Figure 6d—pink curve) and right (Figure 6d—green curve) polynomial curves that estimated the known total $hGRF_{ap}\left(t\right)$ with minimum NRMSE is found. This pair of polynomial fits are considered to be an accurate estimation of the $hGRF_{ap,r}\left(t\right)$ and $hGRF_{ap,l}\left(t\right)$ signals for the current half gait cycle.
  - The difference between the measured (blue curve in Figure 6d) and estimated total $\;hGRF_{ap}\left(t\right)$ (dashed red curve in Figure 6d) curves during the DS phase $Err_{ap}\left(t\right)$, are distributed between the estimated $hGRF_{ap,l}\left(t\right)$ (dashed pink curve in Figure 6d) and $hGRF_{ap,r}\left(t\right)$ (dashed green curve in Figure 6d) signals using Equations (3) and (4) when $t_{G}<t<t_{K}$ and i=ap (Figure 6e).
  - The estimated $hGRF_{ap,l}\left(t\right)$ and $hGRF_{ap,r}\left(t\right)$ signals are multiplied by the weight of the subject and resampled back to the actual length of the measured total $\;hGRF_{ap}\left(t\right)$ signal for the current half gait cycle.
- The next half gait cycle is selected and the estimation procedure described in Step B.II is repeated.

*C.*  *Medial-lateral direction*

**Figure 7:**
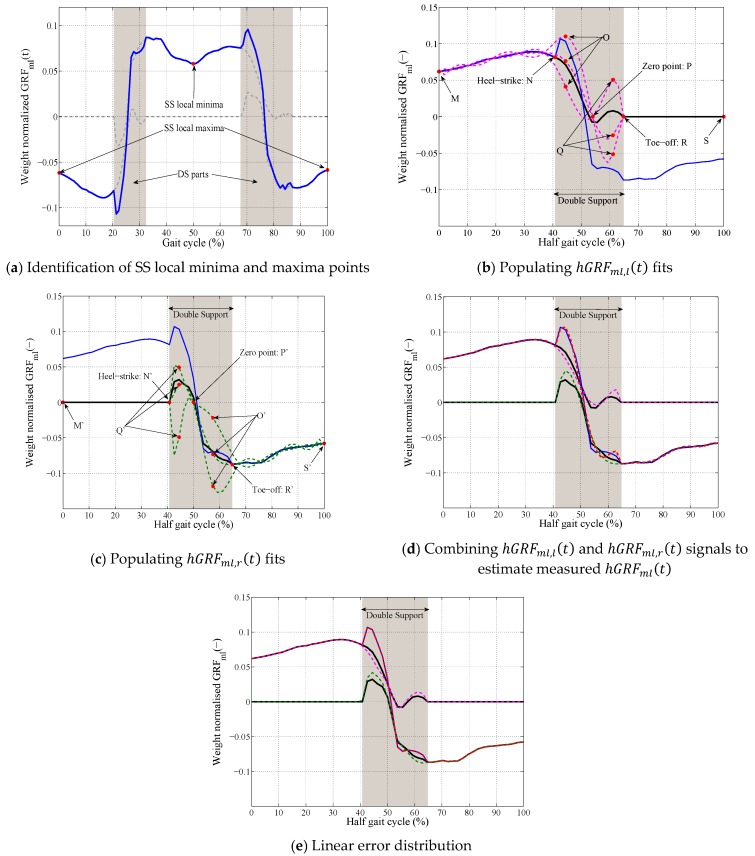
TPM procedure for estimation of $hGRF_{ml}\left(t\right)$

- In the first step, the SS local minima and maxima points are identified from the total $GRF_{ml}\left(t\right)$ (Figure 7a).
- For each segment of the total $GRF_{ml}\left(t\right)$ signal between two consecutive local minima and maxima (total $hGRF_{ml}\left(t\right)$):
  - The total $hGRF_{ml}\left(t\right)$ signal is resampled to 100 points (*T* = 100) and normalized to the weight of the subject (Figure 7b).
  - The timing of the heel-strike ($t_{N}$) and toe-off ($t_{R}$) points for this half gait cycle are identified from the previously-estimated $hGRF_{v,r}\left(t\right)$ and $hGRF_{v,l}\left(t\right)$ signals corresponding to this half gait cycle.
  - A set of polynomial curves of degree nine are generated using the SS part (M-N) and points O, P, Q, and R for the $hGRF_{ml,l}\left(t\right)$ (Figure 7b) where $t_{O}=t_{N}+0.05T$, $t_{Q}=\left(t_{P}+t_{R}\right)/2$, $-0.021<y_{O}<\;0.011$, $-0.032<y_{Q}<0.032$ and $0.42T<t_{P}<0.67T$ (Table 1).
  - A set of polynomial curves of degree nine are generated using the SS part $S^{\prime }-R^{\prime }$, $O^{\prime }$, $P^{\prime }$, $Q^{\prime }$ and $N^{\prime }$ for the $hGRF_{ml,r}\left(t\right)\;$ (Figure 7c) $t_{O^{\prime }}=t_{R^{\prime }}-0.05T$, $t_{Q^{\prime }}=\left(t_{N^{\prime }}+t_{P^{\prime }}\right)/2$, $-0.018<y_{O^{\prime }}<\;0.034$, $-0.032<y_{Q^{\prime }}<0.052$ and $0.26T<t_{P^{\prime }}<0.65T$ (Table 1).
  - A pair of left (pink curve in Figure 7d) and right (green curve in Figure 7d) polynomial curves that estimated the known total $hGRF_{ml}\left(t\right)$ with minimum NRMSE is found. These polynomial fits are considered accurate estimates of the $hGRF_{ml,r}\left(t\right)$ and $hGRF_{ml,l}\left(t\right)$ signals for the current half gait cycle.
  - The difference between the measured (blue curve in Figure 7d) and estimated total $\;hGRF_{ml}\left(t\right)$ (dashed red curve in Figure 7d) curves during the DS phase $Err_{ml}\left(t\right)$, are distributed between the estimated $hGRF_{ml,l}\left(t\right)$ (dashed pink curve in Figure 7d) and $hGRF_{ml,r}\left(t\right)$ (dashed green curve in Figure 7d) signals using Equations (3) and (4) when $t_{N}<t<t_{R}$ and i=ml (Figure 7e).
  - The estimated $hGRF_{ml,l}\left(t\right)$ and $hGRF_{ml,r}\left(t\right)$ signals are multiplied by the weight of the subject and resampled back to the actual length of the measured total $\;hGRF_{ml}\left(t\right)$ signal for the current half gait cycle.
- The next half gait cycle is selected and the estimation procedure described in Step C.II is repeated.

### 3.2. Model Validation

The ultimate goal of TPM is to be used to estimate left and right foot tri-axial $GRF_{i}\left(t\right)$ signals from corresponding total $GRF_{i}\left(t\right)$ signals in real-life environment. Therefore, performance of TPM is assessed for both laboratory and outdoor environment.

#### 3.2.1. Performance of TPM in Laboratory Environment

**Figure 8:**
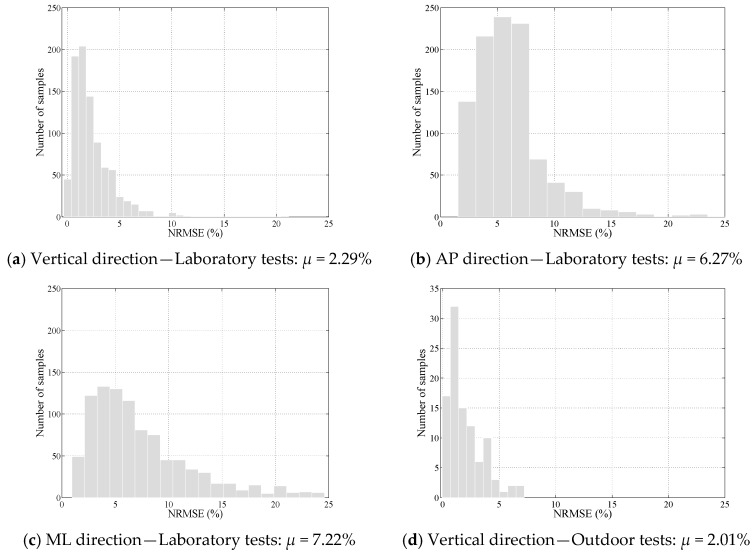
Performance of TPM in the laboratory and outdoor environment.

TPM was used to estimate tri-axial $hGRF_{i,l}\left(t\right)$ and $hGRF_{i,r}\left(t\right)$ signals corresponding to one thousand half gait cycles, randomly selected from the validation dataset of measured $hGRF_{i,j}\left(t\right)$ signals. The NRMSE errors between the measured and estimated $hGRF_{i,j}\left(t\right)$ signals were then calculated and is shown in Figure 8a–c for the vertical, anterior-posterior, and medial-lateral directions, respectively. It was found that TPM estimated the left and right foot $GRF_{v}\left(t\right)$, $GRF_{ap}\left(t\right)$ and $GRF_{ml}\left(t\right)$ signals with mean NRMSE value of *μ* = 2.29%, *μ* = 6.27%, and *μ* = 7.22%, respectively. 

#### 3.2.2. Performance of TPM in Real-Life Environment

The walking gait in real-life environment is characterized with high variability in both magnitude and timing compared with the treadmill-measured $GRF_{i}\left(t\right)$ signals. A set of tests was carried out where 10 subjects walked around the University of Sheffield campus building (in paved urban environment), while wearing a pair of Tekscan F-Scan in-shoe pressure insoles [23] to measure the benchmark $GRF_{i}\left(t\right)$ signals for comparison. The walking pathway was characterized with flat parts as well as uphills and downhills. Subjects were asked to walk normally and no further instructions were given to keep the experiments as realistic as possible. The normal plantar pressures measured under each foot were used to calculate the left and right foot $GRF_{v}\left(t\right)$ signals, and these signals were then summed up to calculate the measured total $GRF_{v}\left(t\right)$. Before and after each trial, subjects walked with their normal speed on the instrumented treadmill while wearing pressure insoles, and the $GRF_{v}\left(t\right)$ signals measured by the treadmill were used to calibrate the pressure insole measurements for each outdoor walking test. This helped to calibrate more accurately the F-scan pressure data, resulting in more accurate estimation of $GRM_{v}\left(t\right)$ signals.

One hundred half-gait cycles were randomly selected from the measured total $GRF_{v}\left(t\right)$ signal and used as input to the TPM method to estimate the corresponding $hGRF_{v,r}\left(t\right)$ and $hGRF_{v,l}\left(t\right)$ signals. Figure 8d shows the NRMSE errors between the measured and estimated $hGRF_{v,r}\left(t\right)$ and $hGRF_{v,l}\left(t\right)$ signals. As it can be seen in this figure, the errors are comparable with the laboratory results with the mean NRMSE value of *μ* = 2.01%. It must be emphasized that, as these NRMSE values are calculated against the $GRM_{v}\left(t\right)$ signals measured by F-Scan as reference, they only represent the errors due to the GRF decomposition process (TPM) and do not include the errors due to the estimation of $GRM_{v}\left(t\right)$ signals using F-scan pressure insoles. Furthermore, the counter-intuitive fact that the real-life mean NRMSE value is marginally lower than the corresponding laboratory value must be interpreted in light of the fact that the benchmark $hGRF_{v,r}\left(t\right)$ signals used to calculate NRMSE values in real-life environment are measured using pressure insoles that has lower high-frequency sensitivity and higher error values compared with the instrumented treadmill measurements.

#### 3.2.3. Application of TPM on IMU Data

Six healthy male subjects (age: 21 ± 1 years, weight: 77 ± 16 kg and height: 1.82 ± 0.08 m) participated in a set of walking gait measurement, where for each subject the treadmill speed was set to 60%, 70%, 80%, 90%, 100% and 110% of their normal walking speed. The tri-axial walking GRF(t) signals pertinent to each foot were recorded using the instrumented treadmill. A set of 12 Opal IMUs [24] were used to measure the tri-axial acceleration and orientation signals at the seventh cervical vertebrae (C7), fifth lumbar vertebrae (L5), upper arms, fore arms, thighs, shanks, and fourth metatarsals with 128 Hz sampling rate. The detailed explanation of the test protocol is presented in [25].

**Figure 9:**
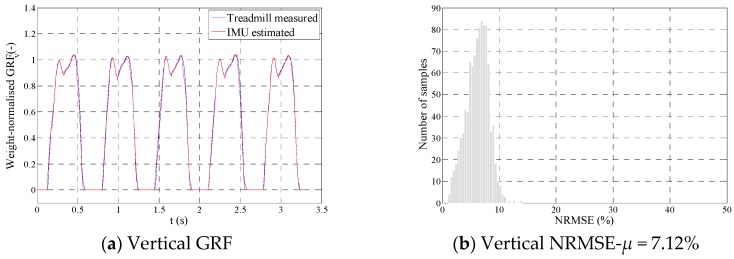

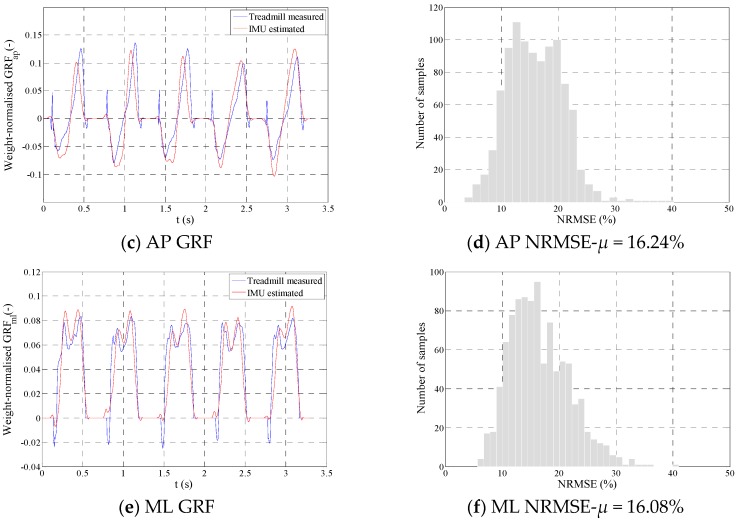
Performance of TPM when applied to inertial measurement units (IMU) data.

Model 2 proposed by Shahabpoor et al. [25] with subject specific training was initially used to estimate the tri-axial total walking GRF(t) signals, only from IMU measurements at C7, L5, and one of the thighs. TPM was then used to estimate tri-axial $hGRF_{i,l}\left(t\right)$ and $hGRF_{i,r}\left(t\right)$ signals corresponding to one thousand half gait cycles, randomly selected from these IMU-estimated total GRF(t) signals (Figure 9a,c,e). The mean NRMSE errors between the treadmill-measured and IMU-estimated $hGRF_{i,j}\left(t\right)$ signals were found equal to $\mu_{v}$ = 7.12%, $\mu_{ap}$ = 16.24%, and $\mu_{ml}$ = 16.08%, for the vertical (Figure 9b), anterior-posterior (Figure 9d), and medial-lateral (Figure 9f) directions, respectively. 

These NRMSE values represent the errors due to both estimation of total GRF(t) signal using Model 2 and estimation of each foot GRF(t) using TPM method and the NRMSE pertinent to each method cannot be computed independently. Using the $\mu_{v}$ = 7%, $\mu_{ap}$ = 13% and $\mu_{ml}$ = 13% NRMSE values reported in [25] for Model 2, decomposing the total GRF(t) into left and right foot GRF(t) using TPM method has increased the total NRMSE values by only $\mu_{v}$ = 0%, $\mu_{ap}$ = 3%, and $\mu_{ml}$ = 3% which are favorably comparable with the NRMSE values reported in Section 3.2.1 and Section 3.2.2 for TPM. 

## 4. Discussion

The accuracy of the results of the TPM method cannot be directly cross-compared with the literature due to fundamental differences in methodology. The NRMSE values reported in [15,20] correspond to the estimation of separate-feet $GRF_{i}\left(t\right)$ signals directly from full kinematic measurements, and the methods suggested in [16,17,18,19] use measured CoP(t) as input. 

However, the TPM methodology is developed using the most extensive experimental dataset presented so far in the literature, and the model is validated both in indoor and outdoor environments. The similarity of NRMSE values for indoor and outdoor environments confirms that the model is versatile and can estimate separate-feet $GRF_{i}\left(t\right)$ signals for time-varying walking speeds and stride length in outdoor environments. The method, furthermore, only needs the total $GRF_{i}\left(t\right)$ signals as input and therefore can be used with wearable IMU systems for monitoring separate-feet $GRF_{i}\left(t\right)$ signals in real-life environment. The TPM however is developed and validated based on the data from young and healthy subjects. The measured data were also limited to smooth laboratory and outdoor walking surfaces.

## 5. Conclusions

In the present study, a method called Twin Polynomial Method was proposed based on a uniquely extensive dataset of measured walking ground reaction forces, to estimate the tri-axial left and right foot walking ground reaction forces from their corresponding total $GRF_{i}\left(t\right)$. The polynomials of degree five, eight, and nine were found to be the best candidates to fit right and left foot $GRF_{i}\left(t\right)$ signals in the vertical, anterior-posterior, and medial-lateral directions, respectively. Results of the TPM method both in the laboratory and in real-life environment showed an average normalized RMSE error of less than 2.5%, 6.5% and 7.5% in the vertical, anterior-posterior, and medial-lateral directions, respectively, compared with treadmill/pressure insole measurements. Further investigation is required for different pathological gaits and for walking on rough terrains to identify the required adjustments to the method.

## 6. Declarations

**Ethics approval and consent to participate:** The investigations were carried out following the rules of the Declaration of Helsinki of 1975 (https://www.wma.net/what-we-do/medical-ethics/declaration-of-helsinki/), revised in 2008. Ethics approval for the walking tests was issued by the ethics committee of the Faculty of Engineering, University of Sheffield, and all subjects provided an informed consent in accordance with the University of Sheffield ethical guidelines for research involving human participants.

**Consent for publication:** All subjects provided an informed consent in accordance with the University of Sheffield ethical guidelines for publication of the anonymized test data.

**Availability of data and material:** The GRF dataset analyzed during the current study are not publicly available as most of the measurements were done as part of confidential industrial projects and authors are not the owners of the dataset. However, a small subset of the data could be available from the corresponding author on reasonable request.

## Figures and Tables

**Table 1:** Twin polynomial method (TPM) polynomial parameters.

$hGRF_{i,j}\left(t\right)$	n	Main Points	Guide Point/s	t	y
$hGRF_{v,r}\left(t\right)$	5	A	-	$t_{A}:\;$SS local minima	Total $GRF_{v}\left(t_{A}\right)$
		B	-	$\mu -2\sigma <t_{B}<\mu +2\sigma ;$*μ* = 39.12; *σ* = 6.00;	Total $GRF_{v}\left(t_{B}\right)$
		C	-	$\mu -2\sigma <t_{C}<\mu +2\sigma ;$*μ* = 68.89; *σ* = 7.78;	$y_{C}\;=\;0$
		-	D	$t_{D}\;=\;t_{C}+0.1T$	$\mu -2\sigma <y_{D}<\mu +2\sigma ;$*μ* = 0.79; *σ* = 0.58;
		E	-	$t_{E}:\;$SS local minima	$y_{E}\;=\;0$
$hGRF_{i,l}\left(t\right)$	5	A	-	$t_{A}:\;$SS local minima	$y_{A}\;=\;0$
		B	-	$\mu -2\sigma <t_{B}<\mu +2\sigma ;$*μ* = 39.12; *σ* = 6.00;	$y_{B}\;=\;0$
		C	-	$\mu -2\sigma <t_{C}<\mu +2\sigma ;$*μ* = 68.89; *σ* = 7.78;	Total $GRF_{v}\left(t_{C}\right)$
		-	D’	$t_{D^{\prime }}\;=t_{B}-0.1T$	$\mu -2\sigma <y_{D^{\prime }}<\mu +2\sigma ;$*μ* = 0.79; *σ* = 0.58;
		E	-	$t_{E}:\;$SS local minima	Total $GRF_{v}\left(t_{E}\right)$
$hGRF_{ap,r}\left(t\right)$	8	F	-	$t_{F}:\;$SS zero crossing	$y_{F}\;=\;0$
		G	-	$t_{G}:\;$Heel-strike of leading foot	Total $GRF_{ap}\left(t_{G}\right)$
		-	H	$t_{H}\;=\;t_{G}+0.05T$	$\mu -2\sigma <y_{H}<\mu +2\sigma ;$*μ* = 0.06; *σ* = 0.02;
		-	I	$\mu -2\sigma <t_{I}<\mu +2\sigma ;$*μ* = 56.04; *σ* = 4.58;	$y_{I}\;=\;0$
		-	J	$t_{J}\;=\;\left(t_{I}+t_{k}\right)/2$	$\mu -2\sigma <y_{J}<\mu +2\sigma ;$*μ* = −0.01; *σ* = 0.01;
		K	-	$t_{K}:\;$Toe-off of trailing foot	$y_{K}\;=\;0$
		L	-	$t_{L}:\;$SS zero crossing	$y_{L}\;=\;0$
$hGRF_{ap,l}\left(t\right)$	8	F	-	$t_{F}:\;$SS zero crossing	$y_{F}\;=\;0$
		G’	-	$t_{G^{\prime }}:\;$Heel-strike of leading foot	$y_{G^{\prime }}\;=\;0$
		-	H’	$t_{H^{\prime }}\;=\;t_{K^{\prime }}-0.05T$	$\mu -2\sigma <y_{H^{\prime }}<\mu +2\sigma ;$*μ* = 0.02; *σ* = 0.06;
		-	I’	$\mu -2\sigma <t_{I^{\prime }}<\mu +2\sigma ;$*μ* = 33.07; *σ* = 6.28;	$y_{I^{\prime }}\;=\;0$
		-	J’	$t_{J^{\prime }}\;=\;\left(t_{I^{\prime }}+t_{G^{\prime }}\right)/2$	$\mu -2\sigma <y_{J^{\prime }}<\mu +2\sigma ;$*μ* = 0.03; *σ* = 0.02;
		K’	-	$t_{K^{\prime }}:\;$Toe-off of trailing foot	Total $GRF_{ap}\left(t_{k^{\prime }}\right)$
		L	-	$t_{L}:\;$SS zero crossing	$y_{L}\;=\;0$
$hGRF_{ml,r}\left(t\right)$	9	M	-	$t_{M}:\;$SS local minima	Total $GRF_{ml}\left(t_{M}\right)$
		N	-	$t_{N}:\;$Heel-strike of leading foot	Total $GRF_{ml}\left(t_{N}\right)$
		-	O	$t_{O}\;=\;t_{N}+0.05T$	$\mu -2\sigma <y_{O}<\mu +2\sigma ;$*μ* = −0.005; *σ* = 0.008;
		-	P	$\mu -2\sigma <t_{P}<\mu +2\sigma ;$*μ* = 54.70; *σ* = 6.32;	$y_{P}\;=\;0$
		-	Q	$t_{Q}\;=\;\left(t_{P}+t_{R}\right)/2$	$\mu -2\sigma <y_{Q}<\mu +2\sigma ;$*μ* = 0; *σ* = 0.016;
		R	-	$t_{R}:\;$Toe-off of trailing foot	$y_{R}\;=\;0$
		S	-	$t_{S}:\;$SS local maxima	$y_{S}\;=\;0$
$hGRF_{ml,l}\left(t\right)$	9	M’	-	$t_{M^{\prime }}:\;$SS local minima	$y_{M^{\prime }}\;=\;0$
		N’	-	$t_{N^{\prime }}:\;$Heel-strike of leading foot	$y_{N^{\prime }}\;=\;0$
		-	O’	$t_{O^{\prime }}\;=\;t_{R^{\prime }}-0.05T$	$\mu -2\sigma <y_{O^{\prime }}<\mu +2\sigma ;$*μ* = 0.008; *σ* = 0.013;
		-	P’	$\mu -2\sigma <t_{P^{\prime }}<\mu +2\sigma ;$*μ* = 45.50; *σ* = 9.67;	$y_{P^{\prime }}\;=\;0$
		-	Q’	$t_{Q^{\prime }}\;=\;\left(t_{N^{\prime }}+t_{P^{\prime }}\right)/2$	$\mu -2\sigma <y_{Q^{\prime }}<\mu +2\sigma ;$*μ* = 0.010; *σ* = 0.021;
		R’	-	$t_{R^{\prime }}:\;$Toe-off of trailing foot	Total $GRF_{ml}\left(t_{R^{\prime }}\right)$
		S’	-	$t_{S^{\prime }}:\;$SS local maxima	Total $GRF_{ml}\left(t_{S^{\prime }}\right)$

## Abbreviations

The following abbreviations are used in this manuscript:

A, B, C, D, E, and A’, B’, C’, D’, E’	Guiding points related to $hGRF_{v,j}\left(t\right)$ (Figure 3a)
ap	Anterior-posterior direction
CoP(t)	Trajectory of center of plantar pressure
DS	Double-support phase of the walking gait
$Err_{i}\left(t\right)$	Error in estimation of the total $hGRF_{i}\left(t\right)$ i∈{v,ap,ml} (Equation (2))
F, G, H, I, J, K, L and F’, G’, H’, I’, J’, K’, L’	Guiding points related to $hGRF_{ap,j}\left(t\right)$ (Figure 3c)
Total $GRF_{i}\left(t\right)$	Total (right and left foot) walking force i∈{v,ap,ml}
Left $GRF_{i}\left(t\right)$	Left foot walking force i∈{v,ap,ml}
Right $GRF_{i}\left(t\right)$	Right foot walking force i∈{v,ap,ml}
$hGRF_{i,j}\left(t\right)$	Left and right foot $GRF_{i}\left(t\right)$ signals corresponding to half of a gait cycle i∈{v,ap,ml} and j∈{l,r}
IMU	Inertial Measurement Unit
M, N, O, P, Q, R, S and M’, N’, O’, P’, Q’, R’, S’	Guiding points related to $hGRF_{ml,j}\left(t\right)$ (Figure 3e)
ml	Medial-lateral direction
n	Order of the polynomial curve
NRMSE	Peak-to-peak normalized root mean square error (Equation (1))
$\mu_{NRMSE}$	Mean value of NRMSE values
$\sigma_{NRMSE}$	Standard deviation of NRMSE values
$P_{n}\left(t\right)$	Polynomial of degree *n*
pdf	Probability Distribution Function
$S_{j}\left(t\right)$	Error scaling function j∈{l,r} (Equations (5) and (6))
SS	Single-support phase of the walking gait
T	Length of the resampled $hGRF_{i,j}\left(t\right)$ signals (T=100)
$t_{k}$	Timing of guiding points (resampled to 100 points) $k\in \left\{A-E,\;A^{\prime }-E^{\prime }\right\}$
TPM	Twin Polynomial Method
v	Vertical direction
$y_{k}$	Normalized amplitude of guiding points $k\in \left\{A-E,\;A^{\prime }-E^{\prime }\right\}$
